# Circulating Placental Vesicles Carry HLA-DR in Pre-Eclampsia: A New Potential Marker of the Syndrome

**DOI:** 10.3389/fimmu.2021.717879

**Published:** 2021-09-03

**Authors:** Chiara Tersigni, Donatella Lucchetti, Rita Franco, Filomena Colella, Caterina Neri, Laura Crispino, Alessandro Sgambato, Antonio Lanzone, Giovanni Scambia, Manu Vatish, Nicoletta Di Simone

**Affiliations:** ^1^Unità Operativa Complessa di Ostetricia e Patologia Ostetrica, Dipartimento di Scienze della Salute della Donna e del Bambino e di Sanità Pubblica, Fondazione Policlinico Universitario A. Gemelli, Istituto di Ricovero e Cura a Carattere Scientifico (IRCCS), Rome, Italy; ^2^Dipartimento Universitario di Medicina e Chirurgia Traslazionale, Università Cattolica del Sacro Cuore, Rome, Italy; ^3^Istituto di Clinica Ostetrica e Ginecologica, Università Cattolica del Sacro Cuore, Rome, Italy; ^4^Unità Operativa Complessa di Ginecologia Oncologica, Dipartimento di Scienze della Salute della Donna e del Bambino e di Sanità Pubblica, Fondazione Policlinico Universitario A. Gemelli Istituto di Ricovero e Cura a Carattere Scientifico (IRCCS), Rome, Italy; ^5^Nuffield Department of Women’s & Reproductive Health, University of Oxford, Oxford, United Kingdom; ^6^Department of Biomedical Sciences, Humanitas University, Milan, Italy; ^7^Istituto di Ricovero e Cura a Carattere Scientifico (IRCCS) Humanitas Research Hospital, Rozzano, Milan, Italy

**Keywords:** human leukocyte antigen, syncytiotrophoblast extracellular vesicles, placenta, pregnancy, pre-eclampsia

## Abstract

**Background:**

Pre-eclampsia (PE) is a common disorder of pregnancy that usually presents with hypertension and proteinuria. The clinical presentation arises from soluble factors released into the maternal circulation from the placenta owing to the stress of syncytiotrophoblast, consequence of defective placentation occurring in the first half of pregnancy. Reduced tolerance of the semiallogeneic fetus by the maternal immune system has been proposed as first trigger leading to poor placentation. We previously observed aberrant expression of human leukocyte antigen (HLA)-DR molecules in the syncytiotrophoblast of a subset of women with PE. Aim of this study was to investigate abnormal expression of circulating HLA-DR in syncytiotrophoblast-derived extracellular vesicles (STBEVs) in women with PE compared to normal pregnant women.

**Methods:**

peripheral venous blood was collected from 22 women with PE and 22 normal pregnant women. Circulating STBEVs were collected by ultra-centrifugation (120000 g) and analyzed for the expression of HLA-DR and placental alkaline phosphatase (PLAP), a specific marker of the placenta, by Western blot analysis and flow cytometry.

**Results:**

circulating STBEVs positive for HLA-DR were observed in 64% of PE women while no HLA-DR positivity was detected in any of the controls (P<0.01).

**Conclusions:**

Aberrant expression of HLA-DR in circulating STBEVs is specifically associated to PE. Further studies are required: a) to define the role of aberrant placental expression of HLA-DR molecules in the pathogenesis of PE; b) evaluate a possible application of detecting circulating HLA-DR positive STBEVs in the diagnosis and prediction of PE in the first and second trimester of pregnancy.

## Introduction

Pre-eclampsia (PE) is a common disorder of pregnancy that usually presents with hypertension and proteinuria. It complicates 3–5% of all pregnancies and remains a major cause of severe maternal and newborn morbidity and mortality worldwide (1). The clinical presentation arises from factors released into the maternal circulation from the placenta as result of syncytiotrophoblast stress. The latter is secondary to defective placentation occurring in the first half of pregnancy. According to the “two stage model”, reduced tolerance of the semi-allogeneic fetus by the maternal immune system could be a potential first trigger in the pathogenetic cascade leading to poor placentation and PE (2). Consistent with this hypothesis, in a previous study, we observed aberrant expression of human leukocyte antigen (HLA)-DR molecules in the syncytiotrophoblast of placentas obtained from women with PE (3).

HLA- DR is a class II molecule constitutively expressed on professional antigen presenting cells (APC) to present exogenous antigens to T cells to elicit antigen specific immune response. In inflammatory conditions the expression of HLA class II molecules can be induced but the possibility that also trophoblast cells might express these antigens is still debated (4, 5).

Tight control of HLA class I and class II expression in villous and extra-villous trophoblast (VT and EVT, respectively) is essential for successful pregnancy outcome (6). In particular, the lack of HLA class II (-DP, -DQ and -DR) molecule expression on trophoblasts prevents maternal T cell allo-immune responses against paternal-derived antigens.

Previously, we demonstrated that aberrant expression of HLA-DR antigen in trophoblast cells can be found in about 40% of syncytiotrophoblast-derived extracellular vesicles (STBEVs), obtained from PE women by dual placental perfusion (3). The positivity for HLA-DR of STBEVs, found in a subset of PE cases, was confirmed by immunohistochemistry on placental sections. In particular, HLA-DR was expressed in syncytiotrophoblast, the cell type releasing the STBEVs in maternal circulation *in vivo* and representing the main maternal-fetal interface in the second half of pregnancy (6).

Here we assessed whether HLA-DR aberrant expression might be confirmed in STBEVs collected from the peripheral blood of women with clinical diagnosis of PE.

## Materials and Methods

### Patients and Sample

This study has been designed and conducted according to the principles of the Declaration of Helsinki and approved by the Ethics Committee of the Università Cattolica del Sacro Cuore of Rome, Italy. Written informed consent was obtained from all recruited individuals.

All women enrolled in this study were selected among those referring to the High Risk Pregnancy Unit and to the delivery suite of the Gemelli Hospital of Rome. For the identification of cases of PE we referred to the definition of the International Society for the Study of Hypertension in Pregnancy (ISSHP) (7). Control patients were recruited among those attending the Gemelli Hospital as outpatients for pregnancy routine medical examinations. Women with diabetes, obesity (BMI >30), pre-existing hypertension, autoimmune or infectious diseases or fetal abnormalities were excluded from control group. All cases and controls were matched for gestational age.

3 ml of venous blood was collected by venipuncture from the antecubital fossa in a tube with EDTA. Each sample was centrifuged twice at 3000 g for 30 minutes at 4°C, to obtain plasma, and then frozen in 500 μl aliquots at -80°C until use.

### STBEVs Collection From Serum

Plasma samples (500µl), previously stored at -80°C, were allowed to attain room temperature. Each sample was diluted (1:1 vol/vol) with PBS and spun at 120,000 g for 90 minutes at 4°C in an Optima XPN ultracentrifuge (Beckman Coulter). After the ultracentrifugation step, the supernatant was discarded and the pellet, which comprised of extracellular vesicles of different sizes, was washed with PBS and spun one more time at 120,000 g for 90 minutes at 4C°. The supernatant was then discarded and the pelleted EVs were then re-suspended with 100 µl of PBS. The protein concentration of STBEVs was determined by Bradford assay, prior to storage at −80°C.

### Western Blotting

For PLAP expression analysis, STBEVs samples collected from sera from 4 preeclamptic and 4 control patients were lysed on ice for 30 minutes in SDS-PAGE sample buffer containing protease inhibitor cocktail (Roche Diagnostics,Basel, Switzerland). Protein content was normalized by loading 20 μg of protein for each sample. Samples were boiled and centrifuged at 13,000g for 10 minutes prior to separation by SDS/PAGE (Invitrogen) and semi-dry transfer to PVDF membrane (Biorad, Hercules, CA, USA). Non-specific binding was blocked with TBS-T (20 mM Tris/HCl, 137 mM NaCl, 0.1%Tween-20, pH 7.6) containing 5% of milk (Santa Cruz Biotechnology Inc., Dallas, Texas, USA) for 1 hour at R/T. Membranes were incubated at 4°C overnight with the NDOG2 antibody (1µl/ml) syncytiotrophoblast-specific mouse monoclonal antibody that recognizes placental alkaline phosphatase (PLAP) (8) in Tris-buffered saline and 0.05% Tween 20 (TBS-T) containing 1% BSA. Membranes were then washed in TBS-T, before incubation with the appropriate (rabbit or mouse) horseradish peroxidase-conjugated secondary antibody (1:4000; Dako, Glostrup, Denmark) for 1 hour at R/T. The antibody used was diluted in blocking buffer. After washing, blots were treated with an enhanced chemiluminescence system (PierceTM, Thermo Fischer Scientific, Waltham, MA USA) and exposed to Hyperfilm ECL (GE Healthcare Life Sciences, Cleveland, Ohio, USA). Densitometric analysis of Western blot was carried out using NineAlliance software (Uvitec Alliance, Cambridge, UK).

### Flow Cytometry Analysis of STBEVs

Analysis of STBEVs was carried out by multi-color flow cytometry, using a CytoFLEX S cytometer (Beckman Coulter) equipped with violet laser (405 nm) excitation sources. This instrument is able to collect SSC (side scatter) off the blue laser (BSSC) and the violet laser (VSSC). The flow cytometer was calibrated using the Megamix-Plus FSC beads emitting FITC of different sizes (100, 300, 500, and 900 nm), as described elsewhere (9). STBEVs number was measured using the cell-counting feature of the instrument that relies on a calibrated peristaltic pump for sample delivery. To distinguish intact vesicles from cell debris, STBEVs were stained with CellTrace™ Calcein Violet (ThermoFisher Scientific, Waltham, Massachusetts, USA), according to manufacturer instructions, as previously described (9).

Prior to running STBEV samples, filtered PBS was also analyzed in triplicate for 2 min to assess the level of background contaminating events (about 100 events/second). To confirm placental origin, STBEVs were stained with anti-Placental Alkaline Phosphatase (PLAP) NDOG2 mouse monoclonal antibody commercially conjugated to Phycoerythrin (PE) (Biolegend UK Ltd., Cambridge, UK) or its PE-conjugated IgG1 control (Biolegend). PLAP is exclusively expressed in placental tissues and commonly used to distinguish vesicles released by the placenta from those coming from other cell types. PLAP positive vesicles were normalized to calcein positive events. Vesicles collected from culture medium of a colorectal cancer cell line were used as a negative control for PLAP positivity. The presence of HLA-DR on STBEVs was investigated by the binding of fluorescein isothiocyanate (FITC)-conjugated mouse monoclonal anti-HLA-DR antibody (clone L243-Abcam) or IgG1 control (Abcam) antibody. Prior to use, all antibodies were filtered through Nanosep 0.2 μm centrifugal devices (Pall Life Sciences) to minimalize interference by background particles. All antibodies were also titrated to ensure their use at the optimum concentration. Fluorochrome compensation for multicolor STBEV flow cytometry was set-up using BD CompBeads (BD Biosciences) labelled with fluorescence conjugated antibodies.

All samples were then blocked with 0.2 µm filtered Fc receptor blocker (10 µL; Miltenyi) for 10 minutes at 4°C before the addition of either filtered PBS (unlabeled control), fluorescence conjugated IgG control (IgG1-PE); or fluorescence conjugated antibody (NDOG2-PE or HLADR-FITC). Tubes were incubated for 1 hours at 4°C in the dark. Stained samples were then made up to 500 µL with filtered PBS and acquired immediately on the flow cytometer (Beckman Coulter). Gates were firstly set so that ≤1% of STBEVs stained positive in the appropriate negative controls. Data were acquired and analyzed by the CytExpert 2.2™ software (version 2.2, CytoFLEX S, Beckman Coulter, Milano, Italy).

### Statistical Analysis

Clinical characteristics were analyzed using unpaired t-test or Chi-square, according to type of variables. The results of the experiments, expressed as the mean ± standard deviation (SD), were analyzed using unpaired t-test performed with Prism software version 9.0. For all analyses, p < 0.05 was considered significant.

## Results

### Clinical Characteristics of the Study Population

Peripheral venous blood was obtained by venous puncture from 22 women with diagnosis of PE and 22 normal pregnant (NP) women with uncomplicated pregnancies, matched for gestational age. Clinical characteristics of PE and NP subjects are shown in **Table 1**. PE women were more frequently nulliparous (p < 0.5) and displayed significantly lower gestational ages at delivery, neonatal birth weights (p < 0.0001) and birth weight percentile (p < 0.0001) compared to NP. As expected, women with PE were characterized by higher blood pressures (p < 0.0001) and detectable proteinuria (p < 0.01) compared to NP women. No significant differences were found in terms of maternal age between PE and control groups. 52% of women with PE were primiparous versus 31% of controls.

**Table 1:** Clinical characteristics of pre-eclampsia and normal pregnant women enrolled.

	Age (years)	GA delivery (weeks)	Nulliparous(%)	Birth weight (g)	Percentile (°)	IUGR(%)	Max SBP (mmHg)	Max DBP (mmHg)	Proteinuria (g/L)
**Pre- eclamptic** (n=22)			59 (n=13)			55 (n=12)			
Average	35.04	31.18		1507.14	25.14		174.38	118.00	3.00
SD	± 6.04	± 5.35		± 941.89	± 25.42		± 16.42	± 10.15	± 3.58
**Normal pregnant** (n=22)			23 (n=5)			0 (n=0)			
Average	32.00	39.41		3327.64	52.03		111.86	71.35	0.00
SD	± 4.97	± 2.11		± 372.58	± 29.60		± 4.35	± 7.28	± 0.00
**P**	0.12	**<0.0001**	**0.01**	**<0.0001**	**<0.0001**	**<0.001**	**<0.0001**	**<0.0001**	**<0.001**

GA, gestational age at delivery; Max SBP, maximum systolic blood pressure; DBP, diastolic blood pressure; IUGR, intrauterine growth restriction.

Values in bold are statistically significant.

### STBEVs Can Be Detected in Peripheral Blood of Pregnant Women by Flow Cytometry

**Figure 1:**
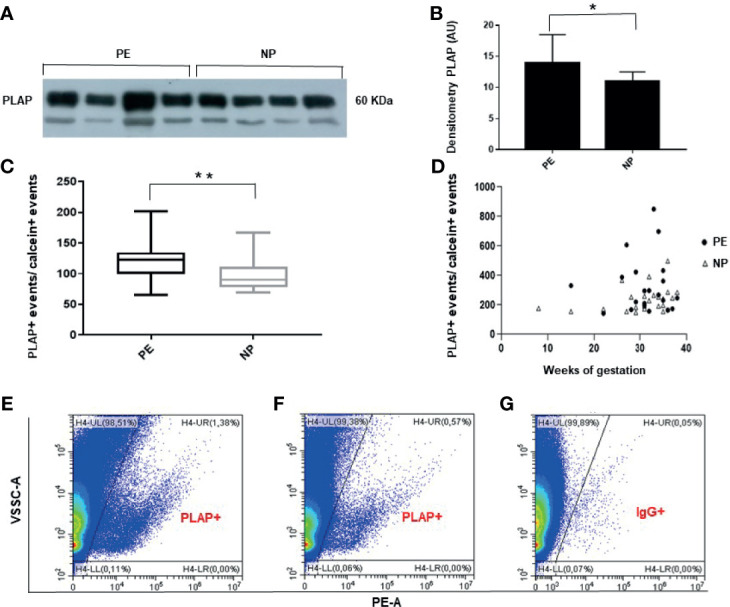
**(A)** Representative Western blot showing PLAP positivity of STBEVs collected by ultracentrifugation of plasma from 4 PE and 4 NP women. The PLAP positive band identifies a significant content of STBEVs in all plasma samples analyzed. **(B)** Histogram showing densitometric analysis of immunoblots. Results are expressed as mean ± SD of 3 experiments. AU: arbitrary units. **(C)** Box plot showing significant higher average levels of PLAP positive (+), corresponding to STBEVs, detected in plasma of PE compared to NP women. PLAP positive events have been normalized to calcein positive (+) events. **(D)** Scatter plot showing STBEVs concentrations detected in both PE and NP women according to gestational age. **(E–G)** Representative flow cytometric analysis for PLAP positivity (+) of vesicles obtained from plasma of a PE **(E)** and a NP **(F)** woman. Levels of STBEVs (PLAP+ vesicles) in PE **(E)** were higher than in NP **(F)**. **(G)** Isotype IgG control used to assess the specificity of anti-PLAP antibody binding to STBEV in the same PE lady of panel **(E)**. NP, normal pregnant; PE, pre-eclamptic women; PLAP, placental alkaline phosphatase; STBEVs, syncytiotrophoblast-derived extracellular vesicles. *p < 0.5; **p < 0.01.

Western blot analysis of STBEVs obtained by plasma ultracentrifugation, performed to assess the presence in maternal circulation of detectable placental-derived vesicles, revealed a clear protein band corresponding to placental alkaline phosphatase (PLAP), a specific marker of syncytiotrophoblast. These results confirmed the presence of significant quantity of STBEVs in the plasma of both PE and NP women (**Figures 1A, B**). Vesicles collected from culture medium of a colorectal cancer cell line, and used as a negative control for PLAP positivity, showed, as expected, no positivity for PLAP (data not shown). Flow cytometric analysis, performed to quantify serum STBEVs content, showed that, according to available literature (10–12), levels of circulating STBEVs were significantly higher in women with PE compared to normal pregnant women (**Figures 1C–G**), although STBEVs levels showed a high standard deviation (SD) and a skewed distribution among the study groups. Interestingly, STBEVs were detectable in the maternal circulation from the early first weeks of gestation (**Figure 1D**). No significant differences were found in terms of calcein positive events or protein content between PE and NP women in vesicles samples collected by plasma ultracentrifugation (data not shown).

### Circulating STBEVs From PE Women Carry HLA-DR

**Figure 2:**
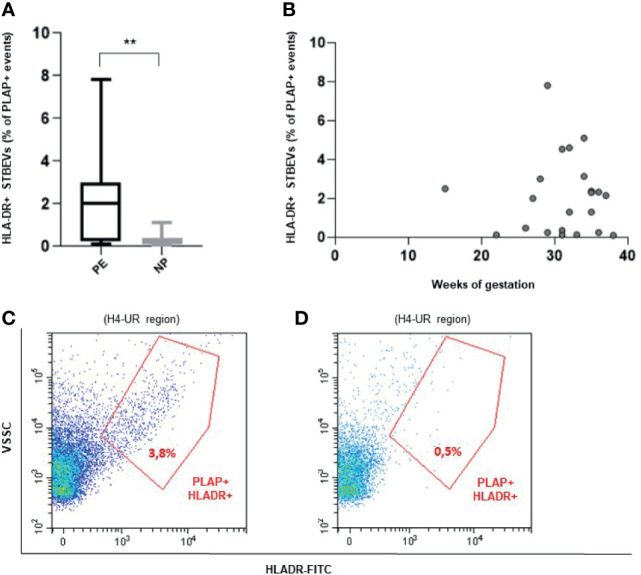
**(A)** Box plot showing a significantly higher percentage (%) of HLA-DR positive (+) STBEVs (PLAP positive events) detected in plasma of PE versus NP women. **(B)** Scatter plot showing distribution of HLA-DR positive (+) STBEVs detected in PE according to gestational age. **(C, D)** Representative flow cytometric analysis for PLAP and HLA-DR double positive (+) vesicles collected from plasma of a PE **(C)** and a NP **(D)** woman. NP, normal pregnant women; PE, pre-eclamptic women; PLAP, placental alkaline phosphatase; STBEVs, syncytiotrophoblast-derived extracellular vesicles. **p < 0.01.

Flow cytometric analysis revealed that HLA-DR positive circulating STBEVs can be detected in PE but not in NP women (p<0.01) (**Figures 2A–D**). In particular, we found a subset of 14 of 22 PE (64% of all PE women analyzed) with aberrant expression of HLA-DR on circulating STBEVs. None of NP women showed detectable positivity for HLA-DR in circulating STBEVs. These data were consistent with the results previously observed on STBEVs collected by dual placental perfusion (3). Interestingly, HL-DR was detectable on STBEVs at all gestational ages analyzed (from a very rare case of PE at 15 weeks until term of gestation) (**Figure 2B**).

When comparing clinical characteristic of PE women with STBEVs carrying HLA-DR with those with HLA-DR negative STBEVs, no significant differences were found (**Table 2**).

**Table 2:** Clinical characteristics of pre-eclamptic women according to STEVs positivity for HLA-DR.

	Age (years)	GA(weeks)	Nulliparous(%)	Birth weight (g)	Percentile (°)	IUGR(%)	Max SBP (mmHg)	Max DBP (mmHg)	Proteinuria (g/L)
**HLA-DR+** (n=14)			64 (n=9)			50 (n=7)			
Average	34.76	33.86		1722	18		165	112	3.78
SD	± 6.59	± 3.79		± 931	± 20		± 14	± 10	± 4.12
**HLA-DR-** (n=8)			50 (n=4)			63 (n=5)			
Average	35.50	31.00		1406	22		168	110	2.32
SD	± 4.84	± 5.39		± 918	± 25		± 16	± 6	± 0.88
**P**	0.75	0.16	0.12	0.46	0.70	0.57	0.65	0.82	<0.48

GA, gestational age; Max SBP, maximum systolic blood pressure; DBP, diastolic blood pressure; IUGR, intrauterine growth restriction.

## Discussion

In this study, we observed that HLA-DR molecules can be detected in STBEVs from 64% of PE women analyzed and that it is technically interrogatable by flow cytometry, through the collection of only 3 ml of peripheral venous blood. Although no significant association between clinical features of the syndrome (i.e. gestational age at onset, association with fetus growth restriction) have arisen, the small sample size does not permit definitive conclusions and further work is needed.

There are findings that are promising, namely: a) HLA-DR specificity in PE: – NP women do not display this protein in the syncytiotrophoblast-; b) the high prevalence of positivity for this protein in about 60% of PE cases investigated; c) the ability to detect this marker in circulating STBEVs by using a minimally-invasive liquid biopsy; d) the potentiality of early detection of HLA-DR positive STBEVs in the first trimester. Nonetheless, the small sample of women analyzed tin this study is a main limitation to get definitive conclusions.

## Conclusions

In this pilot study, we interrogated plasma for STBEVs using the plasma as a “liquid biopsy” of the syncytiotrophoblast in women with clinical diagnosis of PE, to search for HLA-DR aberrant expression in circulating placental STBEVs (corroborated by PLAP positivity). This is made more important when we consider that STBEV usually constitute only about 1% of all circulating vesicles of human blood (most of those are platelet-, endothelium- or leukocyte-derived) (11, 12).

These data are consistent with our previous study showing aberrant expression of HLA-DR in the placenta and in STBEVs obtained by dual placental perfusion from women affected from PE (6). Whether this abnormal expression is a trigger or a consequence of inflammation in the pathophysiology of PE is still an open question. Moreover, whether HLA-DR is maternal or fetal in origin and whether it might be immunogenic to the mother requires further investigation.

In conclusion, HLA-DR is a possible candidate marker worthy to be investigated for its potential application in the prediction and early diagnosis of PE. A well designed multicenter and prospective study is needed to investigate the presence of HLA-DR-positive circulating STBEVs, across the three trimesters of pregnancy, in a larger population of women at high risk of PE. This could answer the question whether finding STBEVs positive for HLA-DR in the circulation might identify women at higher risk of developing PE and the potential predictive value of HLA-DR as a marker of PE.

## Data Availability Statement

The raw data supporting the conclusions of this article will be made available by the authors, without undue reservation.

## Ethics Statement

The studies involving human participants were reviewed and approved by Comitato Etico dell’Università Cattolica del Sacro Cuore, 00168, Rome, Italy. The patients/participants provided their written informed consent to participate in this study.

## Author Contributions

All authors (CT, DL, RF, FC, CN, LC, AS, AL, GS, MV, and NS) contributed to the conception and design of the present study. CT, RF, CN, LC, and NS were involved in the recruitment of patients. DL, CT, and FC performed the experiments. CT, DL, and AS analysed the data. CT, GS, AL, MV, and NS were involved in interpreting the data and critically reviewing the article. All authors contributed to the article and approved the submitted version.

## Funding

This research was supported by a small grant from Fondazione Policlinico A. Gemelli IRCCS (grant number 590002910).

## Conflict of Interest

The authors declare that the research was conducted in the absence of any commercial or financial relationships that could be construed as a potential conflict of interest.

## Publisher’s Note

All claims expressed in this article are solely those of the authors and do not necessarily represent those of their affiliated organizations, or those of the publisher, the editors and the reviewers. Any product that may be evaluated in this article, or claim that may be made by its manufacturer, is not guaranteed or endorsed by the publisher.
